# Brain Tumor Immunotherapy: What have We Learned so Far?

**DOI:** 10.3389/fonc.2015.00098

**Published:** 2015-06-17

**Authors:** Stefaan Willy Van Gool

**Affiliations:** ^1^Department of Microbiology and Immunology, KU Leuven, Leuven, Belgium

**Keywords:** immunotherapy, malignant glioma, dendritic cell vaccines, immunomodulation, galectin-1, oncolytic viruses

## Abstract

High grade glioma is a rare brain cancer, incurable in spite of modern neurosurgery, radiotherapy, and chemotherapy. Novel approaches are in research, and immunotherapy emerges as a promising strategy. Clinical experiences with active specific immunotherapy demonstrate feasibility, safety and most importantly, but incompletely understood, prolonged long-term survival in a fraction of the patients. In relapsed patients, we developed an immunotherapy schedule and we categorized patients into clinically defined risk profiles. We learned how to combine immunotherapy with standard multimodal treatment strategies for newly diagnosed glioblastoma multiforme patients. The developmental program allows further improvements related to newest scientific insights. Finally, we developed a mode of care within academic centers to organize cell-based therapies for experimental clinical trials in a large number of patients.

## Introduction

High grade gliomas (HGG) are brain tumors occurring in adults and children. The WHO grade IV HGG, called glioblastoma multiforme (GBM), is the most frequent brain cancer in adults with an incidence of 3–4 per 100,000 adults per year (1) and 2 per million children (2). The treatment for these patients consists primarily of maximal safe surgery in order to debulk the tumoral mass for symptomatic relief and to obtain tissue for histological diagnosis, followed by radiochemotherapy and maintenance chemotherapy to induce optimal local tumor control. In spite of improved surgery and radiotherapy, and the addition of temozolomide (TMZ) to the multimodal treatment strategy, the prognosis of patients with GBM remains poor: the median overall survival (OS) is about 15 months, with 88% of patients dying within 3 years (3, 4). Relapse is universal and is believed to be due to the extensive spread of tumor cells into surrounding regions of the brain (5, 6). At the time of relapse, the prognosis is particularly poor, with reports of 100% mortality within 18 months (7). A recent review pointed to the progression-free survival (PFS) at 6 month and median OS as most useful and accessible end points, the latter ranging between 5 and 13 months for relapsed GBM patients (8). The prognosis upon recurrence might be improving with the initiation of new multimodal treatment strategies (9–11). Most reports are not yet focusing on long-term survival. In spite of being an orphan disease, the tumor still causes the highest number of years of life lost due to cancer (12). One of the particular challenges with classical chemotherapeutic strategies is overcoming the blood–brain barrier. Therefore, preclinical research is focused on alternate approaches, such as targeted therapy (13) including anti-angiogenesis strategies (14), and especially immunotherapy. Treating cancer by means of immunotherapy (e.g., cancer vaccines, adoptive cell transfer, and checkpoint blockade) has slowly evolved over decades in a nowadays clinically applicable treatment in a number of cancer types (e.g., metastatic melanoma, renal cell carcinoma, non-small cell lung cancer, prostate cancer…).

**Figure 1:**
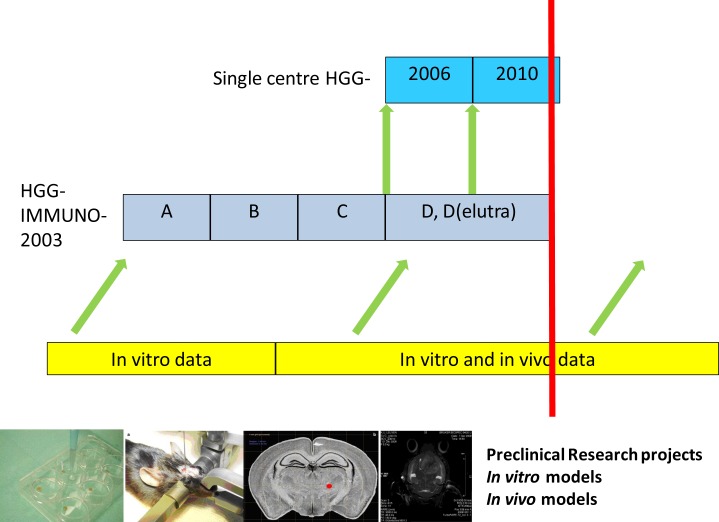
**Immunotherapy for HGG: a translational research program**.

Active specific immunotherapy with autologous mature dendritic cells (DCm) loaded with autologous tumor cell lysate (DCm-HGG-L) is an emerging and innovative treatment approach for patients with HGG. The development of DC therapy in HGG has started in 1999 in our center. Since then, we established a complete translational research program from bench to bed (Figure 1) including *in vitro* experiments (15, 16), *in vivo* experiments in the GL261 model (17–19), early clinical phase I/II clinical trials as part of the HGG-IMMUNO-2003 cohort comparison trial for relapsed HGG patients (20–26), a phase I/II clinical trial HGG-2006 for patients with newly diagnosed GBM (EudraCT 2006-002881-20) (27, 28), and the recently finished phase IIb prospective placebo-controlled double-blind randomized clinical trial (RCT) HGG-2010 (EudraCT 2009-018228-14). In parallel to this clinical program, advanced MRI studies have been performed on HGG, in particular to characterize immunotherapy-related changes (29–32). In this program, insights from preclinical research were translated into the HGG-IMMUNO-2003 cohort (A–D) comparison trial. Data from these cohorts were then used for integration into the multimodal treatment of patients with primary diagnosis of GBM. As such, the vaccination technology from cohort C was used for the HGG-2006 trial, while the technology from cohort D is now used for the RCT HGG-2010. In parallel, according to the evolving legislation, the preparation for the clinical applications was embedded into a Good Manufacturing Practice (GMP) facility within the University Hospitals Leuven. The translation back from bed to bench has been realized by samplings of tumor tissue and blood samples taken at defined vaccination time points. The new preclinical research perspectives in 2014 include galectin-1 targeting as a strategy for immunomodulation and oncolytic virus therapy.

The preclinical and clinical results, together with clinical results obtained independently by other research teams provide a strong rationale to continue exploration of immunotherapy in patients with HGG. We summarized our insights in several reviews and commentary papers (33–39). The emerging field of immunotherapy for HGG has been extensively reviewed by other researchers as well (40–43). A first meta-analysis on the available results in the literature show clear benefit of immunotherapy for OS (44). In this review, it is our intention to focus on our own experience.

## Rationale for Active Specific Immunotherapy Against HGG

### Theoretical concept of dendritic cell vaccination

Dendritic cells (DCs) are a subset of white blood cells, critical to most aspects of adaptive immunity due to their central role as specialized antigen-presenting cells (APCs) in the initiation phase of T cell responses (45). Typically DCs reside as immature cells in almost every organ and tissue at the interface of potential pathogen entry sites. Danger-triggered DCs start to mature: they up-regulate chemokine receptors, which guide them to draining lymph nodes. There, the mature DCs are capable of inducing primary T cell responses due to their high levels of major histocompatibility complex (MHC), adhesion and costimulatory molecule expression. As opposed to the other APC, DCs are able to present and cross-present the antigenic peptides in the context of both MHC Class II and Class I molecules, respectively (46, 47). In this way, they can prime not only CD4+ T helper cells, but also CD8+ cytotoxic T cells (CTLs) (48). Both effector cell types are believed to be necessary to induce an effective cell-mediated immune response (49).

Dendritic cells are not only sentinels in the adaptive immune response, but have also been shown to be strong activators of NK cells and NKT cells (50), thus linking the innate and adaptive immune responses. In this way, both tumor cells with and without expression of MHC class I molecules can theoretically be killed (51). All these particular characteristics make DCs a perfect adjuvant in active specific immunotherapeutic strategies, in which one aims to induce a specific immune response *in vivo* (52–55).

### Justification of the use of dendritic cell technology in glioma therapy

Gliomas have been shown to express an impressive collection of glioma-associated antigens (GAAs) (56). Till today, antigen search is a field of interest (57) including even tumor-driving mechanisms (58). Up till now, however, identification of a universally expressed GAA with a critical downstream cell survival-related function has not been identified. Therefore, just targeting the known GAA using individual peptides would inherently lead to immune escape because of the positive clonal selection of antigen-loss variants (59, 60): those tumor cell clones that do not express the particular, targeted GAA (anymore), will escape from the immune rejection and thus have an important proliferation advantage as compared to the cell clones that do express the targeted GAA. That heterogeneity in GAA expression in gliomas represents the main reason to use whole tumor cell lysates as a source of GAAs to load the DC. In case, the GAAs are expressed not only exclusively on the tumor cells but also on normal healthy cells, tolerance and induction of auto-immunity are possible, both being theoretical hurdles to a beneficial immune response: in the former case, an antitumoral immune response cannot be induced because the GAA is considered a self-antigen and in the latter case, a pathological immune response against normal tissues is mounted.

In general, tumor vaccination strategies are not entirely new anymore (52). Especially for the spontaneously more immunogenic tumors like malignant melanoma (61), renal cell carcinoma (62), mesothelioma (63), leukemia (64), gynecological tumors (65–67) and prostate carcinoma (68), several vaccination strategies have been used in the past. Large-scale production of clinical grade DCs became possible (69), including the development of several closed culture systems to obtain large amounts of DCs for clinical use (70–72). DC vaccination for prostate cancer reached full marketing authorization (Provenge^®^).

The brain, once considered as immune privileged site (73), is a dynamic immunological environment. Astrocytes, microglia and infiltrating immune cells play a major role in the brain during host immunity to antigens (74). The question of immune privilege in the context of malignant glioma is fading (56, 75). Proof of the principle of immunotherapy has been demonstrated in *in vitro* experiments (15, 16) and in several rodent models (37). In these models, induction of protective immunity and immunological memory against syngeneic orthotopic gliomas have been shown after vaccination with DCs loaded with GAAs of different antigen sources.

## Immunotherapy for Patients with Relapsed HGG

### Overview of different cohorts

**Figure 2:**
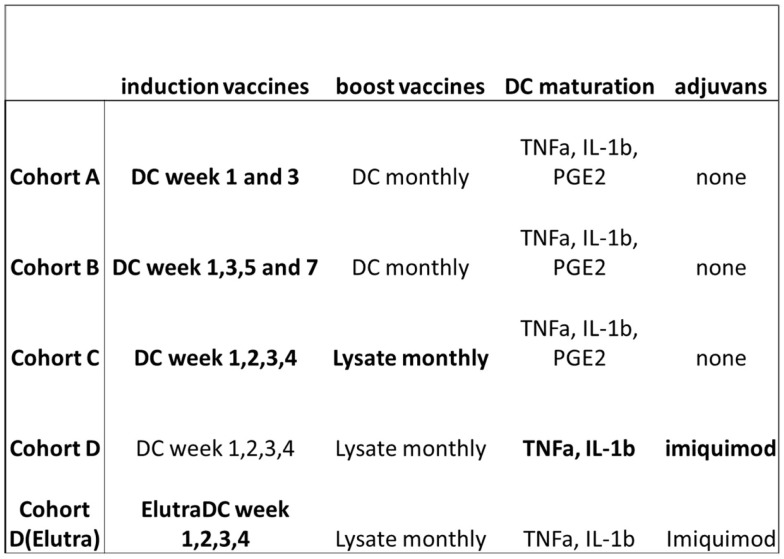
**HGG-IMMUNO-2003 cohorts**.

We started in 2001 to implement preclinical insights into clinical practice after obtaining approval of the local Ethics Committee. Since 2003, we initiated the HGG-IMMUNO-2003 study protocols consisting of sequential therapy-optimalization protocols in consecutive cohorts for patients with relapsed HGG. It is aimed to prove the feasibility and explore the efficacy of immune therapy for HGG, and to “dissect” different aspects of the immune therapy in order to find a putative ideal vaccination strategy. Cohorts have been built up on the most recent insights in vaccination strategy available at time of preparation of the cohort protocol (Figure 2).

- *Cohort A*. The DC vaccination schedule existed of five intradermal injections of autologous mature DC loaded with autologous tumor antigens. DC maturation was induced with the classical cytokine cocktail (IL-1b, TNF-a, PGE2). The latter cytokine cocktail was based primarily on the so-called Jonuleit cocktail (76). Already from the beginning, we omitted IL-6 out of the cocktail. IL-6 was known to play a major role in the induction of a Th17 phenotype of T cell response (77). Injections were administered at week 1, 3, 7, 11, 15.
- *Cohort B*. Based on the observations made in the patient group treated according to the vaccination schedule in cohort A, injections with autologous mature DC loaded with tumor-derived antigens were administered at week 1, 3, 5, 7, (9) and further each 4 weeks.
- *Cohort C*. Based on further observations made in the patient groups treated according to both prior vaccination schedules and based on recent insights in *in vivo* models upon priming with DC and boosting with lysate instead of DC (78), patients were treated with 4 weekly DC-HGG-L injections followed by monthly boosting with HGG-L.
- *Cohort D*. In this cohort, we omitted PGE2 out of the maturation cocktail. PGE2 was already long time ago linked to the induction of a DC2-type (79). Because of its importance for the induction mainly of the mobility of DC (80), it was kept in the classical maturation cocktail. However, PGE2 was later-on also shown to induce IDO activity in human DC, thereby creating a tolerizing DC phenotype (81). Moreover, PGE2 upregulated CD25 on DC, as such believed as a marker of strong DC maturation, but a marker, of which was shown that it was shed in the surrounding thereby consuming the IL-2 needed for autocrine T cell activation. Because not-fully maturated DC themselves play a role in tolerance induction (82), we wanted to apply a method to induce with imiquimod *in vivo* DC maturation after injection (83–86). Imiquimod binds to Toll-like receptor 7 and induces strong DC maturation and activation. Moreover, its role in generating immune responses in a preclinical *in vivo* model of HGG has been described (85). Based on this rationale, PGE2 *ex vivo* maturation was replaced by local application of imiquimod to increase *in vivo* maturation and activation of loaded DC. Within this cohort, we switched at a certain time point from the open cell culture methodology toward a closed cell culture methodology. This group of patients was defined as cohort D(e). The monocytes were isolated with Elutra instead of plastic adherence. Elutriation allows for fast and easy enrichment of monocytes within a closed system, and is superior to other GMP-approved methods (87–89). DCs were cultured in VueLife tissue culture bags instead of Falcon culture flasks. The cytokines used for differentiation and maturation were GMP-certified. Finally, four batches of GMP-DCm-HGG-L were produced at the same time, of which the first was injected immediately as vaccine, while the three other batches were frozen until use. For each of the three remaining induction vaccinations, a batch was thawed and washed once before injection. Of note, the open cell culture methodology continued to include children with relapsed HGG, because the closed culture systems could not be applied to the leukapheresis product of children.

### Updated clinical results

Patients suspected of a relapse of HGG, who could be taken into consideration for immunotherapy, were re-operated upon to maximally remove the tumor and in order to obtain tissue as a source of tumor proteins. Part of the tumor was provided for pathology diagnosis, part was placed immediately in a sterile vial, to be stored at −80°C. Because of the large amount of tumor tissue needed for vaccine production, in rare cases it was impossible for the pathologist to unequivocally prove the recurrent pathology: in these cases, radiological evolution and sometimes amino acid PET scan results were consulted to conclude a relapsing, progressive HGG.

Patients with relapsed HGG were entered into the trial. About 40% of the included patients combined or consecutively applied neurosurgery and immunotherapy with other types of treatment like re-irradiation or chemotherapy upon decision of the referring physician. We obtained clinical results from 366 patients (48 children younger than 18 years and 318 adults above the age of 18 years). These patients belong to the “as treated” group from whom also the RPA was estimated and who received new resection and only immunotherapy till the next event. Median PFS of these children and adults were 3.8 and 2.6 months, respectively; median OS was both 10.6 months. Most importantly, the 2-year OS for these patients with relapsed HGG was 20% (SEM = 6) for children and 22% (SEM = 2) for adults. When the subgroup of 33 children and 247 adults with relapsed GBM was taken separately, median PFS was 2.5 months for children and 2.6 months for adults, median OS was 8 and 9.9 months with a 2-year OS of 10% (SEM = 6) and 17% (SEM = 3), respectively. Thirteen percent (SEM = 8) of adults with relapsed GBM remained free of recurrence for more than 18 months, and 10% (SEM = 2) lived longer than 3 years. Although hard to compare with literature data, the tail of the OS curve seems beneficial to data published on repeated re-operations combined with drug-based adjuvant therapies (11). Our data are difficult to compare to published data on PFS and OS upon new chemotherapy (8) or radiochemotherapy (9, 10). To compare future clinical trials, data should be presented according to prognostic models as has been published after radio(chemo)therapy (90) or immunotherapy (25). Moreover, besides PFS at 6 months and median OS, we believe that long-term OS (2 years or more) should also be considered as further outcome of patients with relapsed HGG in the context of immunotherapy.

**Figure 3:**
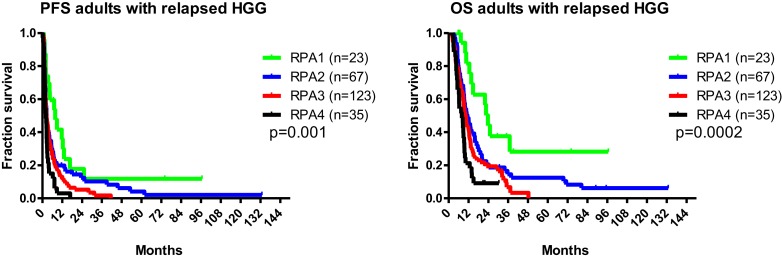
**PFS and OS of adults with relapsed HGG**.

Having included a large series of patients with relapsed HGG and treated with neurosurgery and immunotherapy, it became indeed obvious that clinical risk factors were influencing the prognosis of the patients. This was considered as very important for counseling of the patients and for stratification while designing future RCTs for such patients. Therefore, a novel recursive partitioning analysis (RPA IMMUNO) classification was developed for adults above the age of 18 years with relapsed HGG, and survival data were analyzed on the 117 first included adult patients (25). The RPA classification was based on the age of the patient, the grading of the relapsed tumor (grade III or grade IV), the Karnofsky Self Performance Scale and the estimated mental status. We internally validated the RPA IMMUNO in an extended group of 251 adults with relapsed HGG treated in patient cohorts of the HGG-IMMUNO-2003 protocol and from whom we could retrieve the data for RPA classification. These patients were equally distributed into the four cohorts of patients. Patient characteristics are described in Table 1. As shown in Figure 3, the PFS and the OS of patients belonging to the different RPA risk classes were significantly different.

**Table 1:** **Patient characteristics**.

	HGG-IMMUNO-2003	HGG-2006
Age (median, range)	49 (18–77)	57 (27–70)
Sex (M/F)	161/90	49/28
Grade III/IV/no grading tumors	43/205/3	0/77/0
Number of events (median, range)	2 (2–7)	1
Number of vaccines	6 (4–24)	8 (0–30)
Cohort A/B/C/D/D(e)	11/15/26/72/127	–

The immunotherapy was feasible without major treatment-related toxicities. Almost all patients were treated in an ambulatory setting.

## Immunotherapy for Patients with Newly Diagnosed GBM

### HGG-2006 phase I/II trial

#### Rationale

As next step in our program, we wanted to integrate immunotherapy within the multimodal standard treatment for adults with newly diagnosed and histologically proven GBM (3, 4). A complex rationale was elaborated for the design. (1) Leukapheresis was scheduled after the surgical resection and before radiochemotherapy. After resection of GBM, a functional immune system is normally recovered within 1 week (91). Pro-inflammatory activity after irradiation might influence the activation state of monocytes and hence their differentiation capacity toward DC (92). Moreover, although grade III and IV hematologic toxic effects after radiochemotherapy were minimal (3), mild reduction of the monocyte count cannot be excluded. (2) The four induction vaccines were administered immediately after the radiochemotherapy. The immune suppression after 6 weeks concomitant TMZ was shown to be minimal but still might exist (3). The concept of tumor-specific immunization at time of immune reconstitution after chemotherapy has been demonstrated in several animal models (93, 94) and in clinical practice (95). Moreover, besides the induction of pro-inflammation (92), local radiotherapy might remove suppressor T cells, thus permitting a more effective T cell stimulation *in loco* (96). Another important reason to immunize prior to maintenance TMZ was the finding that the sensitivity of GBM to chemotherapeutics, among which TMZ, after prior vaccination was significantly increased (97, 98). (3) We further continued the boost vaccines during the TMZ maintenance therapy. Injection of lysate-loaded DCs for the priming, followed by boosts with tumor cell lysate alone generated the most effective antitumor effects in a preclinical model. The protocol allowed better CTL responses and also triggered an antitumor humoral response (78). The experiences in cohort C with induction vaccines with DCm-HGG-L and boost vaccines with HGG-L as immunotherapeutic strategy supported the concept for the HGG-2006 trial.

#### Updated Clinical Results

The first aim of this study was to assess the feasibility/toxicity to integrate tumor vaccination within the global treatment plan for an adult patient with newly diagnosed and GBM WHO grade IV, which could at least subtotally be removed. The major primary aim was the PFS at 6 months after diagnosis. To fulfill both the aims of (1) monitoring toxicity (phase I) of this treatment in the newly diagnosed patients and (2) detecting a potential benefit as a treatment strategy (phase II), we included a “STOP and GO” design.

**Figure 4:**
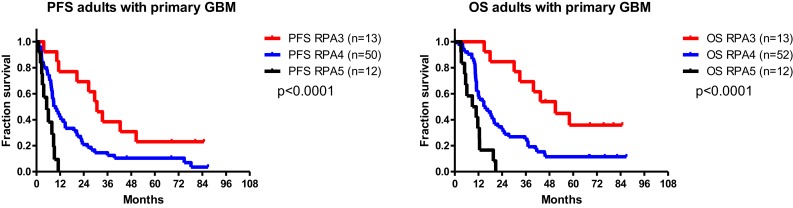
**PFS and OS of adults with primary diagnosis of GBM**.

The results of the pilot phase and the full trial phase have been published recently (27, 28). The trial was feasible without major immunotherapy-related toxicities. The integrated immunotherapy did not affect quality of life. We here present the last updated results (31 July 2014) of the PFS and OS of patients from the HGG-2006 study, divided into the EORTC RPA risk profiles three to five (Figure 4). Patient characteristics are described in Table 1. The data represent the intent-to-treat analysis. The 5-year OS for the EORTC RPA class III and class IV patients was 35.9% (asymmetrical CI95%: +25.4, −24.2) and 11.5% (asymmetrical CI95%: +10.2, −6.9), respectively. As compared to the historical control data of patients belonging to the same EORTC RPA risk profiles (4), patients from EORTC RPA class III had a better OS when immunotherapy was added to the standard treatment. These data were used to power the HGG-2010 trial.

### HGG-2010 prospective placebo-controlled double blind randomized clinical trial

**Figure 5:**
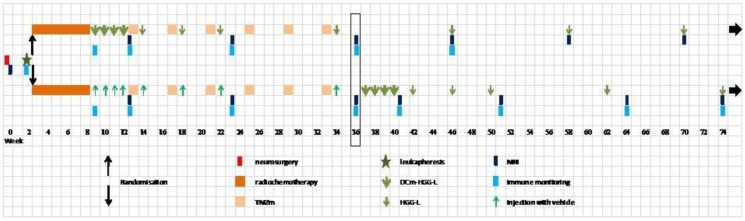
**Outline of the phase IIb randomized clinical trial HGG-2010**.

A prospective placebo-controlled double-blind phase IIb RCT was designed to explore the benefit of immunotherapy as fourth treatment modality to be included within the standard primary treatment strategy for patients with GBM (Figure 5). Supported by our experiences with patients included in HGG-2006, the design of the experimental arm (immunotherapy) is almost similar to HGG-2006. DCm-HGG-L is prepared and maturation is induced similar to Cohort D of the HGG-IMMUNO-2003 trial, using TNF-a, IL-1b, and Imiquimod skin preparation (aimed for TLR7-mediated DC activation). The design of the control arm is the current standard primary treatment: surgery, radiochemotherapy with TMZ, and maintenance chemotherapy with TMZ (3, 4). Randomization is performed with age as stratification variable (99). MGMT (*O*(6)-methylguanine DNA methyltransferase) methylation is not used for stratification. There is emerging evidence that other cytogenetic abnormalities outside MGMT methylation are of strong prognostic value as well (100–102). Primary endpoint of the trial is the PFS after six cycles of maintenance chemotherapy with TMZ. Secondary endpoints are quality of life assessments, OS, and induction of immune responses in both arms.

Patients are unblinded after the assessment of disease status at time of MRI after the sixth cycle of TMZ or at time of progression if earlier progression occurred before the end of the sixth cycle of TMZ. Patients treated in the placebo arm and not yet relapsed (or with a compatible salvage treatment and no steroids after relapse) are treated with the immunotherapy regimen at this later stage, allowing to compare with immunomonitoring early vaccination efficacy during multimodal therapy with late vaccination after multimodal therapy.

The data of this RCT will be subject to the consortium Computational Horizons in Cancer (www.chic-vph.eu) to develop a hypermodel based on granular hypomodels in order to predict for which patient immunotherapy might be of added value. Clinical, radiological, immunological, and molecular data at diagnosis and at early evolution upon the radiochemotherapy will serve as incoming data into the different hypomodels.

## New Preclinical Research Perspectives in 2014

### Targeting galectin-1 as strategy for immunomodulation

#### GL261 Orthotopic Mouse Model

Galectin-1 is a glycan-binding protein which is involved in the aggressive nature of GBM by stimulating angiogenesis, cell migration, and proliferation. In different cancer models, galectin-1 has been demonstrated to play a pivotal role in tumor-mediated immune evasion especially by modulating cells of the adaptive immune system. It was unknown, however, whether the absence or presence of galectin-1 within the glioma microenvironment also causes qualitative or quantitative differences in innate and/or adaptive antitumor immune responses. We explored the role of galectin-1 in the orthotopic GL261 mouse glioma model (19). Stable galectin-1 knockdown was achieved via transduction of parental GL261 tumor cells with a lentiviral vector encoding a galectin-1-targeting miRNA. We demonstrated that the absence of tumor-derived but not of host-derived galectin-1 significantly prolonged the survival of glioma-bearing mice as such and in combination with DC-based immunotherapy. Both flow cytometric and pathological analysis revealed that the silencing of glioma-derived galectin-1 significantly decreased the amount of brain-infiltrating macrophages and myeloid-derived suppressor cells (MDSCs) in tumor-bearing mice. Additionally, we demonstrated a pro-angiogenic role for galectin-1 within the glioma microenvironment. The data provided in this study point to a pivotal role for glioma-derived galectin-1 in the regulation of myeloid cell accumulation within the glioma microenvironment, the most abundant immune cell population in HGG. Furthermore, the prolonged survival observed in untreated and DC-vaccinated glioma-bearing mice upon the silencing of tumor-derived galectin-1 strongly suggests that the *in vivo* targeting of tumor-derived galectin-1 might offer a promising and realistic adjuvant treatment modality in patients diagnosed with GBM.

#### Galectin-1 in the Serum of Patients

In parallel to this preclinical work, we questioned whether increased galectin-1 expression levels were exclusively found at the tumor site or whether galectin-1 could also be detected in the serum of HGG patients. Galectin-1 serum levels were analyzed in a prospective dataset of 43 healthy controls and 125 patients with newly diagnosed or recurrent HGG (103). Samples were taken at the moment of surgical resection and/or 2–3 weeks after surgery. Galectin-1 serum levels were determined using an ELISA for galectin-1. Galectin-1 serum levels depended significantly on age and sex in the control group. Age- and sex-adjusted galectin-1 serum levels were significantly higher in all patient subgroups compared to healthy controls with a high discriminative ability that increased with age. We did not observe a significant decrease in the galectin-1 serum levels upon surgical resection of the tumor. Collectively, the data may represent a first step to establish galectin-1 as a serum biomarker in HGG disease monitoring.

Further longitudinal evaluation is required and ongoing to investigate the value of galectin-1 serum levels in HGG patients as an additional diagnostic marker, but more importantly as a predictor of treatment response and prognosis. Furthermore, galectin-1 serum levels can also provide an important tool for the identification of HGG patients that can benefit from galectin-1-directed therapies that are currently under development.

### Oncolytic virus therapy

The oncolytic features of several naturally occurring oncolytic viruses have been shown on GBM cell lines and in (subcutaneous) xenotransplant models (104). However, orthotopic glioma studies in immunocompetent animals were lacking. We investigated Newcastle disease virus (NDV) in the orthotopic, syngeneic murine GL261 glioma model (105). Seven days after tumor induction, mice were treated intratumorally with NDV. Treatment significantly prolonged median survival of treated animals and 50% showed long-term survival *versus* none in the control group. We demonstrated immunogenic cell death (ICD) induction in GL261 cells after NDV infection, comprising of calreticulin surface exposure, release of HMGB1 and increased expression of PMEL17 cancer antigen. Uniquely, we found absence of secreted ATP. NDV-induced ICD in GL261 cells was shown to occur through programmed necrosis or necroptosis. *In vivo*, elevated infiltration of IFN-γ^+^ T cells was observed in NDV-treated tumors, along with reduced accumulation of myeloid derived suppressor cells. The importance of a functional adaptive immune system in this paradigm was demonstrated in immunodeficient Rag2^−/−^ mice, in which NDV induced a slight prolongation of survival, but failed to induce long-term survival. After secondary tumor induction in mice surviving long-term after NDV treatment, protection against glioma outgrowth was seen in 80% of animals, demonstrating induction of long-term antitumor immune memory after NDV therapy. We thus demonstrated for the first time that NDV has therapeutic activity against GL261 tumors, evidenced in an orthotopic mouse model. The therapeutic effect relies on the induction of a unique ICD route in the tumor cells, which primes adaptive antitumor immunity. The data change the paradigm that the use of oncolytic viruses for anti-cancer therapies should be performed in combination with suppression of potential antiviral immune responses. These insights are of high importance when using oncolytic viruses in combination with tumor vaccines within a multimodal treatment strategy.

## Clinical Experiences on Immunotherapy Obtained in Other Centers

Active specific immunotherapy has been widely studied in many centers in phase I and/or phase II trials. Reviewing 37 reports on DC vaccines between 2000 and 2014, the patient number in each report was in median 15 ranging from 1 to 146. All these trials have been designed in different ways making read-outs hardly comparable. Moreover technologies for the vaccine production and administration routes were different as well. Characteristics of these trials are described in Table 2. Besides, the methodology to perform immune monitoring was variable: DTH tests, relative immune phenotypes of circulating lymphocytes, T cell proliferation and CTL assays, NK cell assays, IFN-γ production (serum, ELISPOT, mRNA expression, FACS), and recent thymic emigrant assay. In spite of all these differences, some general conclusions can be made. Immunotherapy for patients with (relapsed) HGG is feasible, and is safe. Only two immunotherapy-related serious adverse reactions have been reported: an overwhelming inflammatory reaction in a patient with large residual disease (21) and a cutaneous GBM growth after DTH testing of tumor cells which were presumably radio-resistant (106). Induction of autoimmune reactions has not been observed at all, in spite of the fact that crude lysate of tumor tissue used in several trials contained also normal tissue antigens. In most of the trials, an effect is observed being long-term surviving patients and/or immune responses. Immune monitoring data were hardly correlated with clinical data. Most importantly for the further development, a first meta-analysis on the available data shows clear clinical benefit of DC-based immunotherapy for patients with HGG (44).

**Table 2:** **Overview of DC-based clinical trials**.

Study phase	Case report	(20, 148)
	Phase I	(21, 27, 149–161)
	Phase I/II	(22–26, 28, 162–171)
	Phase II	(106, 172)
HGG grade	Grade III	(24, 148)
	Grade III and IV	(23, 25, 106, 149–151, 153, 154, 158, 160, 162, 164–169)
	Grade IV	(20–22, 26–28, 97, 152, 155–157, 159, 161, 163, 170, 172)
Disease status	Relapse (R)	(20–26, 148, 150, 151, 160–162, 165–167, 171)
	New diagnosis (ND)	(27, 28, 97, 149, 152, 155, 156, 159, 169, 170, 172)
	R and ND	(106, 153, 154, 157, 158, 163, 164, 168)
Tumor antigen	Lysate	(20–28, 97, 106, 153, 155, 158, 161–164, 166, 169)
	Peptides	(97, 148, 149, 152, 156, 160, 167, 171, 173)
	Tumor cell mRNA	(151)
	Cancer stem cell mRNA	(159)
	Tumor cell suspension	(154)
	IFN-g-treated tumor cells	(168)
	Apoptotic tumor cells	(170, 172)
	Fusions	(150, 165)
Route	ID	(20–28, 148, 150, 152–154, 156–161, 165)
	SC	(97, 106, 149, 164, 168, 170, 172)
	ID + intratumoral	(162, 166)
	ID + IV	(151)
	Intranodal	(167)

## Modulation to Escape Immune Evasion Mechanisms

There are numerous factors that are responsible for HGG immune evasion (107). Intrinsic mechanisms include low expression of MHC class I and MHC class II molecules on the HGG tumor cells, microglia cells that produce IL-10 and IL-6, and an unbalance of the Th1/Th2 ratio in favor of Th2. Moreover Tenascin-C in the extracellular matrix in glioma prevents efficient immune cell to tumor cell contact. HGG cells produce a lot of immunosuppressive factors like TGF-b and PGE-2. Tumor cells lack costimulatory signals and might induce T cell anergy upon recognition. Moreover, stat-3 expression in the tumor cells promotes tumor immune evasion by inhibiting pro-inflammatory cytokine signaling and by amplifying Tregs. The PD-1L-1 expression on HGG is identified as a strong inhibitor of CD4+ and CD8+ T cell activation. The expression of HLA-E, HLA-G, and the presence of TGF-b and lectin-like transcript 1 are responsible for the absence of an NK attack to HGG. HGG cells express fas and fasL as well as CD70, and produce gangliosides and galectin-1. All these mechanisms are responsible for apoptosis of immune cells. Immune checkpoint blockade in combination with immunotherapy for glioma is therefore an emerging area of research (108). The most important immune evasion mechanisms are, however, the presence of myeloid-derived suppressor cells and especially Tregs.

The presence of Tregs in HGG tumors was found for the first time in 2006 (109). The number of Tregs infiltrating the brain was correlated with the WHO grade of the glioma (110). The suppressive activity of HGG-derived Tregs was demonstrated (109, 111–113). In preclinical research, we clearly showed the role of Tregs not only to block the antitumoral immune response (18) but also to change the inflammatory tumor microenvironment (114). Tregs have been shown to play a role on M2 macrophage differentiation (115) and MDSC functioning (116) in rodents. Tregs are particularly recruited into HGG by the production of CCL2 and CCL22 (117). Moreover, Tregs in HGG patients have a higher expression of the CCL2 receptor CCR4 as compared to controls. In the peripheral blood, a relative increase of the Treg fraction in the CD4 compartment as compared to controls was also described (118). Functional studies on Tregs from HGG patients became possible through isolation and characterization of this population as CD4 + CD127dim cells (119). These clinical data clearly show the presence and function of Tregs within the tumor microenvironment and even systemically.

Treg depletion and Treg inhibition are a widely discussed strategy in cancer (120). TLR ligands have been shown in preclinical models to inhibit Treg function and enhance *in vivo* tumor immunity (121, 122). Also TMZ (117, 123, 124) and gemcitabine (125) have been found to affect Treg infiltration in rodent models. Treatment with Sunitinib (126–128) or low dose paclitaxel (129) decreased the number of Tregs in cancer patients. Specific Treg depletion strategies have been performed in humans with anti-CD25 mAb daclizumab or with IL-2 diphtheria toxin conjugate denileukin diftitox (Ontak) (130–132). Treg depletion and immunological benefits could be obtained, especially with daclizumab. However, a trial had to be stopped because of availability of the product (130). The most important depleting strategy is the metronomic use of CPM (133–140). CPM suppresses *in vitro* induction of Tregs (141). The Treg depleting activity of CPM has been demonstrated in murine models in the context of vaccines (142). Some studies in humans have shown improvement of T cell effector function associated with a reduction in Treg numbers after low dose CPM (135). The timing and dose are critical for a robust CPM-based protocol able to induce significant ablation of Treg inhibitory functions in patients. Because the Treg depletion is aimed to be performed shortly after neurosurgery, potential interaction with used corticosteroids as described in mice should be taken into account (143).

## Toward a New Health Care Model for Advanced Therapy Treatments

Autologous mature DCs loaded with autologous tumor lysate belong to the category of advanced therapy medicinal products (ATMP). According to EU Regulation 2007/1394/EC, ATMP for human use means (1) a gene therapy medicinal product as defined in Part IV of Annex I to Directive 2001/83/EC; (2) a somatic cell therapy medicinal product as defined in Part IV of Annex I to Directive 2001/83/EC; or (3) a tissue engineered product. In that context, DCs differentiated out of monocytes are defined as ATMPs. The boost vaccines consisting of HGG-L are regulated by the Directive 2004/23/EC. ATMPs in academic hospitals can be produced under the hospital exemption clausule. Hospital exemption means preparation of ATMPs on a non-routine basis according to specific quality standards, and used within the same Member State in a hospital under the exclusive professional responsibility of a medical practitioner in order to comply with an individual medical prescription for a custom-made product for an individual patient.

The production and administration of personalized ATMPs together with other anti-cancer therapies in a multimodal treatment approach for very diseased patients should be considered as Advanced Therapy Treatment for these patients, preferentially performed in centers of excellence by fully equipped specialty teams with particular multidisciplinary knowledge on basic, translational, and clinical science around the ATMP within the given clinical context. From the beginning of the translational research program, the working model was organized as a multicentre collaboration. The goal was to make this experimental treatment strategy in clinical trials easily accessible for all potential patients in and outside the country. By doing this, a multiple “win” situation was created: the accessibility to immunotherapy program was easy for each patient, the referring specialist remained involved in the patient care (vaccination in ambulant setting) and in the scientific evolutions of the program, and the vaccination center obtained large series of patients so that experience could be maximized and scientific data generated within short periods. It might take time before patient-specific ATMPs that are used within a very complex clinical context, will reach industrialization for their production. In their report to the European Parliament and the Council in March 2014, the reporters from the European Commission pointed to creating a more favorable environment for ATMP developers working in an academic or non-for-profit setting, including by promoting early contacts with the authorities through the application of the fee reduction for scientific advice and by extending the existing certification scheme to these developers (144). Nevertheless, the DCVax^®^-L vaccine is developed by Northwest Biotherapeutics as an adjunct to the treatment of GBM, and is currently under evaluation in a phase III trial (145).

Obviously, the use of autologous *ex vivo* cultured mature loaded DCs is labor-intensive and expensive. This means a small-scale production for each individual patient as well as an adapted health care model to develop and provide such technologies. Meanwhile, strategies are searched for targeting DCs in the patient themselves. Appropriate pattern recognition receptors ligands are bound to tumor antigens to provide necessary adjuvant immune signals. Antigens are bound to antibodies which target particular receptors on DCs for internalization of the antigen and subsequent presentation (146). Besides antibody-based DC targeting, nanoparticles are rapidly emerging as new vehicles for delivering vaccines. Nanoparticles are a platform for co-encapsulating TLR ligands with the tumor antigen, and for targeting DCs through monoclonal antibodies or carbohydrate ligands (147).

## Conclusion

Immunotherapy for HGG is feasible and has shown promising clinical results in a subgroup of patients without major adverse events. Decisive scientific results from large randomized trials are needed and awaited before the true position of DC vaccination in the therapy of HGG can be established. In parallel, patients who can benefit from this technology are characterized and defined. With current available basic science knowledge, further improvements of techniques and treatment strategies are reachable. However, administrative burdens to produce individualized vaccines remain a major threat, so that research focusses on as much as possible standardized off-the-shelf consumables for their production.

## Conflict of Interest Statement

The author declares that the research was conducted in the absence of any commercial or financial relationships that could be construed as a potential conflict of interest.
